# Examining Intersectoral Action as an Approach to Implementing Multistakeholder Collaborations to Achieve the Sustainable Development Goals

**DOI:** 10.3389/ijph.2022.1604351

**Published:** 2022-05-16

**Authors:** Joslyn Trowbridge, Julia Y. Tan, Sameera Hussain, Ahmed Esawi Babiker Osman, Erica Di Ruggiero

**Affiliations:** ^1^ Division of Social and Behavioural Health Sciences, Dalla Lana School of Public Health, University of Toronto, Toronto, ON, Canada; ^2^ Faculty of Arts and Science, University of Toronto, Toronto, ON, Canada; ^3^ School of Epidemiology and Public Health, University of Ottawa, Ottawa, ON, Canada; ^4^ Institute of Health Policy, Management and Evaluation, Dalla Lana School of Public Health, University of Toronto, Toronto, ON, Canada

**Keywords:** equity, sustainable development goals, intersectoral action, partnerships, healthy public policy

## Abstract

**Objectives:** The Sustainable Development Goals (SDGs) re-orient action towards improving the social and ecological determinants of health and equity. SDG 17 calls for enhanced policy and institutional coherence and strong multi-stakeholder partnerships. Intersectoral action (IA) has a promising history in public health, including health promotion and global health. Some experts see IA as crucial to the SDGs. Yet less is known about how IA is conceptualized and what promising models exist with relevance to the SDGs. We sought to investigate how IA is understood conceptually and empirically.

**Methods:** We conducted a narrative review of global public health and political science literatures and grey literature on the SDGs to identify theoretical models, case studies and reviews of IA research.

**Results:** Multiple competing conceptualizations of IA exist. Research has focused on case studies in high-income countries. More conceptual clarity, analyses of applications in LMICs, and explorations of political and institutional factors affecting IA are needed, as is attention to power dynamics between sectors.

**Conclusion:** IA is required to collaborate on the SDGs and address equity. New models for successful implementation merit exploration.

## Introduction

The Sustainable Development Goals (SDGs), released by the United Nations (UN) in 2015, aim to unite a global agenda for sustainable development and address the gaps of their predecessor, the Millenium Development Goals (MDGs), released in 2000. The MDGs were criticized for excluding low- and middle-income countries (LMICs) in their development and obscuring the interdependence of socio-structural factors that contribute to the poor health of citizens, specifically working and living conditions that made certain populations more vulnerable to illness than others [1–4]. The SDGs represented a way to “do health development differently,” with more locally led and globally supported approaches (1 p. 5). A global consultation process increased participation from LMICs, and the mandate was widened to the “triple helix of sustainable development” which included economic, social, and environmental goals to replace the narrow MDGs (1 p. 3). The new agenda commits to “leave no one behind” by calling upon all nations to eradicate poverty and reduce inequities and by addressing the root causes of discrimination [5]. This includes changing “discriminatory laws, policies and social practices that leave particular groups of people further and further behind” [6].

In the 6 years the SDGs have been in operation, countries have set up governance and monitoring structures to implement the goals nationally and through partnerships and official development assistance funds. In 2017, the UN released a Global Indicator Framework (GIF) to help track progress on each goal, and many countries have created their own national indicator framework using the GIF as a guide. Recognizing that to “leave no one behind” requires a collective, whole of society effort, SDG 17: Revitalize the global partnership for sustainable development, focuses on inclusive partnerships within and between countries. It has 19 targets across finance, technology, capacity building, trade, and a category named “systemic issues.” Within this category, SDG 17 calls for enhanced policy and institutional coherence for sustainable development and strong multi-stakeholder partnerships between public, public-private, and civil society.

The best approaches to carry out the call for enhanced coherence and multistakeholder partnerships for achieving the Goals are the subject of recent research (see [7–9]). The UN has developed the SDG Partnership Guidebook [10] to help organizations understand the building blocks of successful partnerships, from stakeholder engagement to implementation to review and renewal. Other research has focused on public-private partnerships, surfacing best practices and critical considerations for engaging the private sector in sustainable development (see [11–14]). In this paper, we examine the research on intersectoral action (IA) as an approach to achieve coherence and multistakeholder partnerships. We define IA as “the alignment of strategies and resources between actors from two or more policy sectors to achieve complementary objectives.” Intersectoral action has a long history as a promising approach in health promotion and global health, and some experts see IA as crucial to shift the sustainable development agenda beyond the narrow focus of the MDGs towards tackling the structural and social determinants of health [1, 9, 15]. However, relatively less is known about how IA is conceptualized across different literatures and what promising models exist with potential relevance to the SDG agenda.

This paper presents the results from a narrative review of the concept of intersectoral action from the fields of public health and political science that pre-date its uptake for the advancement of the SDGs. Given the promise for IA’s application to the SDGs, we investigate its conceptual origins in public health, including the sub-fields of health promotion and global health, and trace the developments at major international health conferences from the 1970s to the start of the SDGs in 2015. We also review similar conceptual development in the field of political science and its sub-field of public administration. This analytic approach allowed us to explore the development of intersectoral action and study key features of successful models available from empirical case studies in interectoral health interventions. We argue that intersectoral action is necessary for making progress on the SDGs, and that it is valuable to investigate the growing sub-set of literature on intersectoral action to identify underrepresented areas for future research. We concur with other authors that one of these underrepresented areas is how power is theorized in empirical studies, specifically at the level of governance in formal intersectoral collaborations [16–19]. Although the SDG Partnership Guidebook discusses addressing power imbalances and maintaining equity in partnerships (10 pp [50–51]), we find insufficient attention to the power dynamics of intersectoral collaborations in the literature. We maintain that this is a missing aspect of research that is important for understanding how global and national health infrastructures will need to change to advance the SDGs using intersectoral action approaches.

## Methods

This narrative review combines peer-reviewed literature on intersectoral action with peer-reviewed and grey literature on the SDGs. To inform our search strategy, we performed an initial review of UN documents and websites related to the SDGs and literature on intersectoral action purposefully identified by project team members. Our search terms are listed in Table 1. We searched PubMed, limiting results to English language articles published after the year 2000 (when the MDGs were launched). Titles and abstracts were reviewed to capture articles that were theoretical discussions of intersectoral action concepts, models and case studies of intersectoral action, and scoping reviews of intersectoral action research. The results were cross-referenced with previously gathered literature from project team members, and after eliminating duplicates, we included 43 articles.

**TABLE 1:** Search terms (Toronto, Canada. 2022).

Concept 1—Intersectoral action	Concept 2—Sustainable Development Goals	Concept 3—Policy Theory
Intersectoral Action	Sustainable development goals	Public policy
Intersectoral Action for health		Policy theory
Intersectoral Collaboration		Public administration
Intersectoral Cooperation		Governance
Partnerships		
Multi-stakeholder partnerships		
Horizontal partnerships		
Multisectoral Action		
Multisectoral collaboration		
Healthy Public Policy		
Health in All Policies		
Whole-of-government		
Whole-of-society		
Joined-up governance		

## Results

### Origins and Definitions of Intersectoral Action

Intersectoral action has roots in the fields of health promotion and global health, building on research into inequitable health outcomes that illuminated the impact of issues in sectors outside of health. The 1997 World Health Organization (WHO) International Conference on Intersectoral Action for Health used Kriesel’s definition to define IA: “a recognized relationship between part or parts of the health sector with part or parts of another sector which has been formed to take action on an issue to achieve health outcomes or intermediate health outcomes in a way that is more effective, efficient or sustainable than could be achieved by the health sector acting alone” [20]. Related terms include “Health in All Policies” (HiAP), which describes an approach to policy-making that prioritizes health regardless of whether the health sector is involved [21], with a focus on systematically integrating health concerns and developing ongoing collaborative relationships across all government decisions [22]; “multisectoral collaboration,” which describes a process for multiple stakeholders to collaborate for a shared outcome [8], and “cross-sector collaboration,” which describes partnerships that take place across several sectors [23]. We present a summary of the developments in global health in Table 2; many of the concepts related to IA grew from these milestone events [24, 25]. In 2013, the WHO Helsinki Statement on Health in All Policies established a global agreement on the importance of decisions in all sectors on population health.

**TABLE 2:** Key milestones in the development of Intersectoral Action for health (Toronto, Canada. 2022).

1978- International conference on primary health care (Declaration of Alma-Ata)	-Urgently called on all governments to promote the health of all people
	-Recognized the importance of the social determinants of health in primary care
1986- First International Conference on Health Promotion (Ottawa Charter for Health Promotion)	-Set the goal of achieving “Health for All” by 2000
	-Clearly defined health promotion as, “the process of enabling people to increase control over, and to improve, their health”
	-Recognized the need to move into the healthy public policy sector to achieve Health for all
1988- Second International Conference on Health Promotion	-Recognized the importance of governments implementing joint policies across the social, economic and health sectors to advance health promotion
1991- Sundsvall Conference	-Emphasized the link between health and the environment by laying out six strategies for environmental change to promote health
1992- Conference on Environment and Development (Rio Declaration)	-Set of 27 principles to promote sustainable development
	-Recognized the need for environmental legislation to promote healthy lives
1997- Conference on Intersectoral Action for Health	-Clearly defined “intersectoral action for health”
	-Highlighted the importance of collaboration across sectors to solve complex issues in health
1997- Fourth International Conference on Health Promotion (Jakarta Declaration)	-Reaffirmed the importance of the Ottawa Charter, while highlighting new priority areas to achieve health promotion
	-Recognized health literacy as a necessity for health promotion
	-Recognized the need for participation to create change
2000- Millennium Summit	-Lead to the creation of the MDGs
	-MDG 8 sought to develop global partnerships to promote development
2005- 6th Global Conference on Health Promotion (Bangkok Charter)	-A set of goals and actions to address the social determinants of health through health promotion
	-Recognized the inequalities between HICs and LMICs
	-Listed private, non-private, non-governmental and international organizations as important partners to create sustainable development through collaboration
2005- 2005 World Summit	-Follow up to the Millennium Summit
	-Reiterated the commitment to the MDGs
2013- 8th Global Conference on Health Promotion	-Focus of the conference was “health in all policies”
	-Recognized the responsibility that governments have to promote health through the policies that are created
2012-2015—Post 2015/Pre-SDG Development Agenda	- A 3 year process to plan for the end of the MDGs in 2015; resulted in launching the SDGs
	- 2012 UN Conference on Sustainable Development Rio+20 was the first agreement to launch a process to develop the SDGs
	- 2013 UN General Assembly approval of ‘road map’ to SDGs
	- Created the Open Working Group on SDGs
	- Established the High-level Political Forum on Sustainable Development and the Intergovernmental Committee of Experts on Sustainable Development Financing

A parallel conceptual development evolved in the field of public administration in the 1980s and 90s. Research turned towards new ways that different levels and sectors of government could cooperate with each other and collaborate with private and civil society actors. Mondal et al. review models of collaboration developed outside of health promotion, finding that “theory-building on mechanisms of coordination, institutionalization processes and dimensions of culture, values and power has been primarily conducted in political science … and public administration” ([26] p. 2). They highlight that the environmental sciences, in contending with a topic that crosses many sectoral boundaries, have also contributed to processes of governing across sectors. Mondal et al. review the rise of “joined-up-government” and “whole-of-government” ideas, which emerged to address barriers to policy coherence and the silos of separate operating structures entrenched in the public sector. These approaches “focused on building a strong unified set of values and collaboration among public servants” and “the dynamics of interaction between institutions, and ensuring challenges related to control, coordination and accountability” ([26] p. 11). Governance research investigates these interactions. In trying to resolve the “scourges of modern society and its bureaucracy: hyperspecialization, organizational silos, lack of cross-silo engagement” ([24] p. 334), research on collaborative governance and intersectoral action has become a sub-speciality on its own, spurring theoretical and empirical research into models and understanding the effects of political context. A non-health-focused definition of intersectoral action is offered by Dubois et al. as: “working with more than one sector of society to take action on an area of interest to achieve better results than those obtained working in isolation” (20 p. 2939). Sectors noted in their scoping review include public administration, social work, health, education, agriculture, and the environment. They also use the term “sectors” to mean public, private and non-profit organizations. The dual lineage of notions of intersectoral action strengthen its application to governance processes, offering relevance for governments engaging collaboratively on the SDGs [24, 26].

### Conceptualizations and Models of Intersectoral Action

Intersectoral action research considers the fundamental principles, key skills, and necessary conditions for successful collaboration across sectors. The public administration sciences have emphasized macro-level institutional reform, investigating the role of coordination, accountability, and power [26]. Mondal et al. find that an institutional cultural shift away from hierarchy towards “a learning culture with more tolerance for uncertainties … the creation of values and trust, promoting team-building, and establishing a cohesive work culture” (26 p. 13) is a top requirement for collaboration. They list a series of factors that will enable intersectoral action: setting up new formal structures such as interdepartmental committees, and working groups with experts, academics and community leaders; encouraging informal emergent networks to increase communication and build social capacity and reciprocity between actors; ensuring funding, human resources, and technological support are allocated to intersectoral work; and strong leadership and accountability mechanisms. Additionally, policy and public administration research has generated insight into the governance mechanisms, policy frames, subsystems and policy entrepreneurs, and policy instruments for successful collaboration [22, 24, 27, 28].

Theoretical development on IA for health is closely linked to efforts to identify the best approach to tackle the social determinants of health and promote health equity. Much of this research has focused on important macro-level factors for successful collaboration, such as trust among partners. For example, de Montigny et al. propose three main dimensions for success: 1) engagement processes that allow for a common understanding while encouraging a diversity of perspectives; 2) motivation for those involved based on shared values, trust, and frequent communication; and 3) the capacity for collective learning and action through dedicated resources, leadership, expertise, and institutional support [23]. This model emphasizes adaptation to “changing circumstances and unanticipated situations within complex socio-ecological systems” (23 p. 41). Similarly, Shankardass et al. posit that a health-in-all-policies approach requires “an ongoing adaptive process” to ensure collaborations can respond to uncertainties produced in the complex relationships between government actors [29].

Other studies present findings from cases of cross-sector collaborations using interviews with main actors and evaluation data to create lists of facilitators and barriers to success. In 2006, the WHO partnered with the Public Health Agency of Canada (PHAC) to pioneer work and present lessons for future IA research and practice from 15 LMIC case studies. Results found that stability of the socio-political environment, the leadership and will of political and civil society actors, and the number of dedicated resources impacts the success of intersectoral initiatives [30]. A later report (2008) by the same team found that issue framing was important because it affects which partners are invited to the table and how outcomes are defined [31]. They argued that a broader framing of the health issue to include social indicators that go beyond the concept of health equity helped engage sectors outside health and build the case for intersectoral action as a good approach to tackling complex problems. Similarly, Holt recommends emphasizing equity instead of health to engage non-health sectors in action on the social determinants of health [32]. Smith and Weinstock agree, stating, “arguably, intersectoral strategies for health equity by their very nature may limit the opportunity or appetite of non-health sectors to collaborate. This is because intersectoral action on health inequities takes as its starting point the privileging of equity in health over equity for other social goods” (18 p. 1). In the context of the SDGs, a further re-framing is recommended, moving from health equity to sustainability to encourage more intersectoral action [33].

The 2008 PHAC/WHO report also found that IA projects are more challenging at the national level in governments with complex policy environments and shared responsibilities than in more linear divisions of responsibilities for social determinants of health; and “true cooperation in planning, implementation and evaluation was facilitated when it took place at several levels simultaneously” (31 p. 4). A key recommendation was that the role of the health sector should be flexible, only leading intersectoral work if it matches the framing of the identified issue or problem. Subsequent case studies and scoping reviews have identified common success factors, such as building trust among partners, creating connections between different sectors through networks, providing clarity and accountability for roles and responsibilities, information-sharing, engaging the public, and understanding the local political context [22, 23, 29, 34–37]. The UN Department of Economic and Social Affairs (UNDESA) released the SDG Partnership Guidebook, which describes successful partnerships for achieving the SDGs [10]. According to UNDESA, successful partnerships involve mobilizing and optimizing resources to focus on sufficiency rather than scarcity and can include multiple stakeholders and actions to achieve a healthy environment, a thriving society, and a prosperous economy. Research focused explicitly on intersectoral action in LMICs acknowledge that challenges “may be more acute … where institutions are frequently weak, and fragmentation … can undermine coordination” (38 p. 1). These studies highlight the need for high level political commitment, joint agreements for collaboration rather than competition among government agencies, and partnerships with civil society and the private sector (see [38, 39]).

Policy and governance theories have been recently applied to case studies of intersectoral action to investigate why and how it has become a leading approach across high, middle, and low income countries. Mauti et al. applied John Kingdon’s 1984 multiple streams theory of policy change to understand how intersectoral action became the dominant framework for achieving the SDGs in Kenya [40]. Kingdon posited that policy change happens when a window of opportunity is opened through the alignment of three aspects of a political environment—problems, politics, and policies. Each of these streams may be operating independently, but if policy actors and advocates can find opportunities to bring them together, significant change can occur. Mauti et al. show that Kenya’s uptake of the Health in All Policies approach to addressing the SDGs can be understood using Kingdon’s theory. They argue that the burden of diseases that require a social determinants of health approach (problem stream), combined with an increased understanding and application of HiAP in policy offices (policy stream) and new intersectoral governance and implementation bodies created by a Kenya-specific SDG strategic planning process (politics stream), created a window of opportunity for a commitment to HiAP and intersectoral collaboration. Baum et al. apply a different governance theory to understanding what led to a HiAP process in South Australia [41]. They use the “ideas, interests, and institutions” framework that suggests these three factors can explain how policy develops and governance processes come to dominate. Baum et al. rely on policy scholars Exworthy, Kickert and Howlett, among others, to theorize the way HiAP gained traction in South Australia. They concluded that without institutionalized governance mechanisms and evidence that the idea of HiAP is the best approach, it will not be consistently applied in all sectors. Other applied governance and policy theories include actor-network theory in neighbourhood commitees in Montreal, Canada [42, 43] and municipal-level planning and governance processes in Norway and Denmark [44, 45]. More empirical applications of governance and policy theories to explain the rise of intersectoral action as a dominant approach to tackling complex problems are needed, given the attention to intersectoral action as a mode of operating to achieve the SDGs [46, 47].

## Discussion

Efforts to bring lessons from the field of intersectoral action together are increasing, as evidenced by the recent special issue of BMJ Global Health dedicated to health intersectoralism in the SDG era ([9, 48]) and several scoping and bibliographic reviews of the literature on intersectoral action and health ([20, 25, 26, 34, 49, 50]). Experts are also investigating the field of IA research itself. While empirical case studies form the majority of IA research, there is a growing body of conceptual literature that attempts to gather the lessons of successful collaborations to create models of how to create and sustain intersectoral action (see [23]). Recent work also calls for more attention to the role of power relations, dynamics and power asymmetries in collaborations, the variety of governance and other theoretical frameworks that are used, and the development of indicators to measure collaboration that could be transferable across contexts (see 1 [6, 17, 46, 47, 51]).

The role of power dynamics within and between countries is undertheorized in the study of intersectoral action. Glandon et al. analyzed 205 studies of multisectoral collaboration, arguing that the use of case study methods obscures the needs of policy makers and does not always generate transferable insights [17]. 82% of the papers reviewed focused on HICs, 9% on LMICs, and 9% had a global focus. They also found that most studies focused on “implementation tips”; few explored contextual factors that could affect collaboration (17 p. 11). Drawing on this review and the reflections from participants at a 2018 Health Systems Research Symposium in the United Kingdom, Glandon et al. state, “power dynamics between partner institutions are often underexplored. There is limited understanding of how power imbalances affect multistakeholder collaboration formation, structure and implementation, including negotiation between stakeholders about what constitutes “success” and which indicators to measure” (17 p. 12). Macro-theoretical discussions of power are also missing in IA research [18]. For example, an ethical analysis of if, where, when, and how governments should use coercive powers to intervene in the distribution of goods to engineer greater social equity, and how those powers might be at odds with each other from the perspective of different government departments, is needed.

Emerging research on power relations in collaborative processes calls for greater attention to this important issue. Dewulf and Elbers theorize how power is mobilized in cross-sector partnerships, arguing that the closer a partner is to alignment between their institutional field and the issue field of the collaboration, the more power they have to both directly and subtly influence the others involved towards favourable outcomes for themselves [16]. Friel et al. study the power dynamics in public policymaking across seven case studies of IA in Australia [19]. The authors created a health equity power framework, identifying how the types, forms, places and levels of power influenced the development and implementation of multisectoral action. They found that “power dynamics have created problematic situations and maintained the status quo within policies that have been harmful for health equity, but also enabled means of transforming the policy system in ways that are good for health equity” (19 p. 9). Their study revealed that “the socially created rules and mandates, especially associated with neoliberalism, racism, sexism and biomedicalism, guide and constrain policy decision-makers’ choices, through setting the expectations about how the game should be played and who has power in the game” (19 p. 10). They call for a reconfiguration of governmental institutional processes to allow for more voices, specifically public-interest groups, to be included in multisectoral policy activities.

### Limitations

This narrative review synthesized the conceptual and empirical literature on intersectoral action from public health, including health promotion and global health, and political science, including public administration. It is limited by the evolving nature of this literature and by the large and diverse number of terms used to describe intersectoral action, some of which we may have missed in our search and might have been database specific. It was also beyond the scope to analyze factors influencing effective implementation of IA, especially given the recency of the SDGs. Rather, our narrative review sought to critically investigate and reflect on how intersectoral action has been defined and conceptualized in a range of literature inside the broad fields of public health and political science for further consideration as a key approach to achieving the SDGs.

### Conclusion

There has been a steady interest in the application of intersectoral action for health equity and action on the social determinants of health. This interest has grown over the past 50 years through international conferences on global health and has been taken up as an approach specifically with health equity goals in mind. Renewed efforts to engage multiple sectors on the socio-structural determinants of health has emerged with the post-2015 global development agenda, given the significant level of collaboration required to achieve the SDGs. Research has been concentrated on empirical case studies in high-income countries, and calls for more conceptual clarity, analyses of applications in LMICs, and explorations of political and institutional factors affecting intersectoral action continue. Insights can be gained from the literature on public administration, notably the factors that contribute to success inside rigid policy and political environments. Theories of governance and policy-making can inform intersectoral action literature, as shown in the exploration of the “ideas, interests and institutions” and the “policy windows” theories by Baum et al. [41] and Mauti et al. [40], respectively. In both the conceptual and empirical literature, more attention is needed to illuminate the power imbalances that impact how intersectoral action unfolds, and how this affects the goal of health equity. The SDGs’ “leave no one behind” principle, and the focus on “partnerships for the goals” in SDG 17, provide an opportunity to investigate how power operates in different models and processes across different country contexts. Specific attention to how equity is understood and measured in a variety of intersectoral collaborations for the SDGs is needed.
